# Approaches to preventing workplace sexual harassment of nurses or minimising its adverse consequences: a scoping review

**DOI:** 10.1186/s12912-025-04179-2

**Published:** 2025-12-08

**Authors:** Milena Marta Bruschini, Maj Britt Dahl Nielsen, Rahel Naef, Maria Schubert, Tina Quasdorf

**Affiliations:** 1 School of Health Sciences, Institute of Nursing, ZHAW Zurich University of Applied Sciences, Winterthur, Switzerland; 2 Faculty of Medicine, University of Zurich, Zurich, Switzerland; 3 National Institute of Public Health, University of Southern Denmark, Copenhagen, Denmark; 4 Faculty of Medicine, Institute for Implementation Science in Health Care, University of Zurich, Zurich, Switzerland; 5 Centre of Clinical Nursing Science, University Hospital Zurich, Zurich, Switzerland

**Keywords:** Nursing workforce, Occupational violence, Prevention, Scoping review, Sexual harassment, Strategies

## Abstract

**Background:**

Workplace sexual harassment of nurses is a common problem worldwide, influencing nurses’ well-being and the quality of patient care. Widely applicable recommendations and specific guidance for health care organisations are lacking. There is a need for systematically developed and evaluated interventions to prevent and mitigate sexual harassment of nurses. Hence, this study aims to identify existing approaches to preventing sexual harassment of nurses or minimising its adverse consequences at the individual, organisational and network levels to provide a basis for the development of appropriate interventions.

**Methods:**

A systematic scoping review was conducted, involving a systematic search of the literature in four medical databases in July 2024, complemented by a supplementary search including grey literature. All study designs and non-scientific sources referencing empirical or non-empirical research were included if they focused on nurses and approaches to preventing workplace sexual harassment or mitigating its adverse consequences. A thematic analysis was conducted, and a numerical summary was made to categorise the approaches and to quantify their frequency.

**Results:**

Out of 3,912 records identified through database searches, 32 records published between 1984 and 2024 were included. Twelve followed the IMRAD (introduction, methods, results, and discussion) structure and were empirical. Of the twenty non-IMRAD records, only four included empirical data; the rest were theoretical or opinion based. Approaches to preventing sexual harassment of nurses or mitigating its consequences were situated at the individual or organisational levels, but none extended beyond a single organisation to address the network level. The approaches were analysed and categorised into eight domains: (1) ‘organisational culture’, (2) ‘infrastructure and working environment’, (3) ‘leadership’, (4) ‘guidelines’, (5) ‘reporting of incidents, (6) ‘education’, (7) ‘nurses’ approaches to reacting or coping’, and (8) ‘patient-centred approaches’.

**Conclusion:**

A wide range of approaches at the individual and organisational levels are available to prevent sexual harassment of nurses or to minimise its adverse consequences, with education and reporting of incidents being the most frequently mentioned. However, for most of the approaches, research on their effectiveness and feasibility in practice is lacking. To systematically evaluate the approaches identified within different contexts, further research is needed.

**Supplementary Information:**

The online version contains supplementary material available at 10.1186/s12912-025-04179-2.

## Background

Sexual harassment of nurses is a worldwide phenomenon [1, 2]. While the prevalence in studies varies widely (5–95%), the literature agrees that it is a major problem [3–10]. Sexual harassment is a form of sexual misconduct [11] that encompasses any unwelcome behaviour of a sexual or gender-related nature which violates a person’s dignity [12]. It can manifest in various forms, such as verbal, nonverbal, and physical harassment [10]. The decisive factor in defining such behaviour is the perception of the person being harassed, not the intention of the person committing the act [12]. In the context of nursing, sexual harassment can originate from various groups, including patients, patients’ relatives, nurse colleagues, supervisors, and physicians. The primary perpetrators vary across studies and are influenced by the cultural context and setting [2, 5, 9, 13, 14]. Studies from the Global North indicate that sexual harassment is most frequently perpetrated by patients [5, 14–16]. In contrast, in the Global South, the main perpetrators are more likely to be physicians, patients’ relatives, or nurse colleagues [7–9, 13, 17].

Compared with other health care professionals, nurses are more often affected by workplace sexual harassment [18–20]. Several factors contribute to this increased vulnerability. First, nursing tasks often involve close physical contact with stressed, intoxicated, or cognitively impaired individuals [13, 15, 20]. Second, nurses work in hierarchical settings with possible power imbalances [5, 17, 21]. Third, the gender composition of the nursing workforce may further elevate the risk. The profession remains predominantly female [22], and some studies suggest that female healthcare workers are at higher risk of experiencing sexual harassment, often linked to stereotypical perceptions of women in caring roles [5, 23–25]. However, other studies indicate that male nurses may be disproportionately affected [1]. This may be explained by the unbalanced gender ratio within the profession, which increases the risk of sexual harassment against the minority group [5]. This demonstrates that nurses, regardless of gender, are at increased risk of experiencing sexual harassment due to structural and role-related factors inherent in the profession [1, 5, 23–25]. In summary, workplace environments characterised by close physical contact, limited privacy, dissatisfaction, as well as underlying stereotypes, gender, or power imbalances may create conditions that facilitate inappropriate behaviour, such as sexual harassment [5, 15, 17, 18].

The adverse consequences for nurses, institutions and patients are considerable. Approximately 70% of affected nurses report psychological and physical challenges [7], such as anxiety, humiliation, headaches, and exhaustion [5, 7, 13, 15, 25, 26]. Additionally, several studies have confirmed the negative impact of workplace sexual harassment on work performance, motivation, and quality of care [5, 7, 16, 26]. Sexual harassment, particularly when experienced on a regular basis, may result in nurses leaving the profession [7, 13, 15, 16, 27, 28]. Nonetheless, widely applicable interventions and specific guidance for health care organisations are still lacking [25, 29–32]. Efforts are needed not only to prevent sexual harassment, but also to mitigate its negative consequences, as even the strongest engagement cannot entirely eliminate its occurrence [3, 21].

According to Mirzoev et al.‘s conceptual framework [33], sustainably improving health care systems requires a multi-level approach involving interventions at the individual, organisational, and network levels, where the network level refers to interorganisational collaboration. Although some studies document actions related to sexual harassment of nurses, they are rarely evaluated in terms of their usefulness or suitability for prevention or mitigation [5, 8, 15, 30]. Moreover, the approaches reported often target only a single level and tend to be educational in nature, focusing primarily on raising awareness, risk assessment skills, building resilience, or teaching de-escalation techniques [34].While a few systematic reviews have addressed workplace violence in health care and its prevention, such as the ongoing systematic review by Song et al. [35] with no published results, the published systematic review by Kumari et al. [34], and the recently published meta-analysis by Abdulwehab and Kedir [36], none have specifically focused on preventive measures against sexual harassment of nurses. Although these reviews include sexual violence and nursing within their scope, they do not focus on nurses’ perspectives or synthesise targeted prevention strategies specifically for sexual harassment. Furthermore, these systematic reviews are limited to experimental, quasi-experimental, and pre-post studies but exclude qualitative research and non-empirical methods such as literature overviews, opinion pieces, and commentaries [34–36].

Evidence-based nursing involves using the best available research-based information, along with contextual knowledge, empirical knowledge and internal and external consumer needs and preferences [37]. Consequently, evidence-based knowledge should not be limited to randomised trials or meta-analyses [37]. This broader understanding is particularly relevant when addressing a multifaceted, context-dependent, and sensitive issues such as sexual harassment of nurses, where research on specific approaches to prevention and mitigation of adverse consequences remains limited. The complexity of the issue indicates that a single intervention alone cannot address the problem, and various approaches at different levels are needed to combat sexual harassment in everyday nursing care [30, 33, 38, 39].

### Aim and rationale

To date, there is no systematic overview that maps the knowledge landscape on all approaches to preventing sexual harassment of nurses or mitigating its adverse consequences. Therefore, the aim of this scoping review is to identify approaches to combating sexual harassment of nurses or its adverse consequences at the individual, organisational and network levels. It includes approaches for nurses worldwide, of all genders, working in any care setting, and considers sexual harassment perpetrated by any group. While these diverse contexts are considered, the review does not aim to compare variations between subgroups. Rather, this work maps the current state of the literature and the types of evidence available.

In this scoping review, approaches to combating sexual harassment of nurses are defined not only as evidence-based interventions derived from empirical studies but also as strategies described in other research types and non-scientific sources, such as expert opinions, recommendations, and guidelines. Incorporating these additional evidence types into practice is useful when scientifically sound knowledge is still lacking [40, 41].

## Methods

### Design

This study follows the design of a systematic scoping review and adheres to the updated methodological guidance provided by the Joanna Briggs Institute (JBI) [42, 43]. In contrast to a systematic review, the literature is not used to examine the feasibility, appropriateness, efficacy or effectiveness of interventions. Rather, a scoping literature method serves to identify the types of evidence available and possible gaps in knowledge regarding preventive measures to counter sexual harassment of nurses or to minimise its adverse consequences. On the basis of this indication, Munn et al. [44] recommend conducting a scoping review rather than a systematic review. This manuscript was reported in accordance with the PRISMA-ScR (Preferred Reporting Items for Systematic reviews and Meta-Analyses extension for Scoping Reviews) guidelines, as outlined by Tricco et al. [45].

### Protocol and registration

The study protocol of this systematic scoping review [46] was published on the Open Science Framework (OSF) in June 2024 (OSF Registry Number: 3qapf). Given the lack of evaluated evidence-based interventions and the abundance of literature based on non-scientific methods, the research team decided to deviate from the protocol in the following aspect: the research question was adapted, and the term ‘evidence-based interventions’ was replaced with ‘approaches’. This allowed for an expansion of the scope and the inclusion of literature based on non-scientific methods and grey literature. However, this adaptation made it necessary to expand the exclusion criteria to ensure a minimum standard of source reliability. As a result, informal literature, such as newspaper articles and expert opinions, was excluded if it did not refer to any empirical or non-empirical research.

### Eligibility criteria

The inclusion and exclusion criteria were defined using the PCC (population, concept, context) mnemonic of Peters et al. [43, 47], along with additional criteria such as design, language, and year of publication (see Table 1). All listed criteria had to be met for inclusion.

**Table 1:** In- and exclusion criteria

Tool & Criteria	Inclusion	Exclusion	Definition
PCC: Population	Nurses	Other health care professions, family caregivers, informal caregivers	Nurses refers to professional nurses across all genders and qualification levels, such as advanced practice nurses, registered nurses, licensed practical nurses, nursing assistants, and student nurses. Nurses must explicitly be mentioned and make up the largest proportion of the population studied.
PCC: Concept	Sexual harassment, sexual abuse, sexual assault, sexual violence	Any other type of workplace violence	This study focusses specifically on violence with a sexual reference. Sexual harassment must explicitly be mentioned as part of workplace violence and results presented separately.
PCC: Concept	Every kind of approach to prevent sexual harassment or mitigate its consequences on any level (individual, organisational and network)	No focus on approaches	This study creates an overview on all possible approaches already described against workplace sexual harassment of nurses at any level described by Mirzoev et al. [33].
PCC: Context	All nursing settings, work related	Private life	The study focuses exclusively on the work context of nursing professionals and includes all possible perpetrator groups that nurses may encounter in this setting.
Other: Design(s)	All research types and non-scientific methods (e.g. guidelines, commentary papers, expert opinions)	No reference to any empirical or non-empirical research	Literature of all study designs and non-scientific approaches are included, if referring to literature.
Other: Language	German, English	Any other language	All published Literature in German and English are considered, as the authors are fluent in these languages.
Other: Publication year	All	No restriction	All published literature up until 30. June 2024 was included without any restrictions on publication year.

Note. PCC (Population, Concept, Context) rule by Peters et al. [43, 47]

### Search strategy

The search strategy was developed according to the PCC mnemonic of Peters et al. [43, 47]. While the concept dimension of the PCC mnemonic was covered by two search strings, the authors decided not to integrate the context dimension into the search string. The reason is that existing studies on sexual harassment of nurses have focused mostly on workplace contexts. Therefore, the search included three search strings:

Search string 1 (Population): Nurses.

Search string 2 (Concept Part I): Sexual harassment.

Search string 3 (Concept Part II): Approaches to preventing or minimising consequences.

The search strings with suitable keywords and mesh terms were combined with the Boolean operators AND and OR, as well as with proximity operators. In addition, truncations (*) were used to search for all endings of a word stem. The search fields ‘title’ and ‘abstract’ were searched without applying any date restrictions. The search strings were developed collaboratively within the research team, based on the review objective and relevant literature. To ensure quality and comprehensiveness, the final search strategy was reviewed by two independent research librarians.

The search was conducted in July 2024 in the electronic databases MEDLINE (via Ovid), CINAHL Ultimate (via EBSCO), PsycInfo (via EBSCO), and Cochrane (via the Cochrane Library). Additionally, a supplementary search was performed in Google Scholar, care magazines, trial registries, evidence-based guidelines, and grey literature platforms and through forward and backward citation tracking of the included records. Details on the final search strategy per database and the supplementary search can be found in the metadata (Supplementary Material 1).

### Study selection

Records identified through the database search were imported into EndNote 20 and deduplicated in line with Bramer et al. [48]. The remaining records were then imported into Covidence software [49]. Titles and abstracts were independently screened by two reviewers (MMB, TQ) based on the inclusion and exclusion criteria. Discrepancies in ratings were discussed between the two reviewers, and if no consensus was reached, the respective references were discussed with a third reviewer (MS). The same strategy was applied for the full-text screening, except that, in addition to the general inclusion and exclusion criteria, records were excluded if the full text could not be found despite extensive searches in databases, Google, Google Scholar, and university library resources or if retrieving them would have required unreasonable resources.

Due to resource restrictions, the supplementary search was conducted by a single researcher (MMB), who screened titles exclusively. In Google Scholar and grey literature platforms, only the first 10 pages (approximately 100 records per search) were screened because of the high volume of hits. Titles deemed relevant based on inclusion and exclusion criteria were further evaluated by reviewing the full texts. The included records were then reviewed by a second researcher (TQ) to confirm their inclusion.

### Data extraction and management

Information about the methods and metadata of each record, including the author(s), title, year of publication, country of origin, nursing setting, and study design or record type, was extracted on the basis of the JBI criteria of Peters et al. [47]. Additionally, whether the record followed the IMRAD (introduction, methods, results, and discussion) structure was noted. The IMRAD format is a widely accepted standard for reporting original research articles in medical peer-reviewed journals and is typically associated with a structured and methodologically transparent presentation [50, 51]. This classification was used to provide an indication of the general scientific orientation of the records. The distinction serves descriptive purposes only and does not constitute a formal quality appraisal. Moreover, information regarding approaches to preventing sexual harassment of nurses or minimising its adverse consequences was extracted and categorised according to the three capacity levels (individual, organisational and network) of Mirzoev et al.‘s conceptual framework [33]. Information about the ‘testing status’ of approaches was also extracted to indicate the degree of application and evaluation in practice. This rating was developed inductively by the authors and comprises three categories: (1) ‘recommended’ – approaches that were suggested but without indication of actual application or evaluation in practice; (2) ‘applied in practice’ – approaches reported as being used in nursing workplaces but without information on formal evaluation; and (3) ‘evaluated’ – approaches that have been assessed for their effectiveness or utility in practice. One researcher (MMB) categorised the testing status, and a second (TQ) reviewed the allocation for consistency.

The data charting process was conducted by one researcher (MMB) and verified by a second researcher (TQ). In line with the scoping review methodology, no critical appraisal of the included records was conducted [44].

### Data analysis and synthesis

After the data were extracted, a thematic analysis was conducted, and a numerical summary made, as described by Arksey and O’Malley [52], in stage 5. This stage involves collating, summarising, and reporting the results and providing an overview of the included records [52]. The content was organised thematically to identify approaches addressing sexual harassment and potential research gaps, followed by a basic numerical analysis to map the extent, nature, and distribution of both the records and the identified approaches.

The data and text analysis software MAXQDA [53] was used for this purpose. The reported approaches to preventing sexual harassment or minimising its adverse consequences were categorised by inductive coding and then thematically grouped into domains by one researcher (MMB). The codes and domains were then cross-checked by another reviewer (TQ). The identified domains were subsequently summarised descriptively.

## Results

### Selection of sources of evidence

**Fig. 1:**
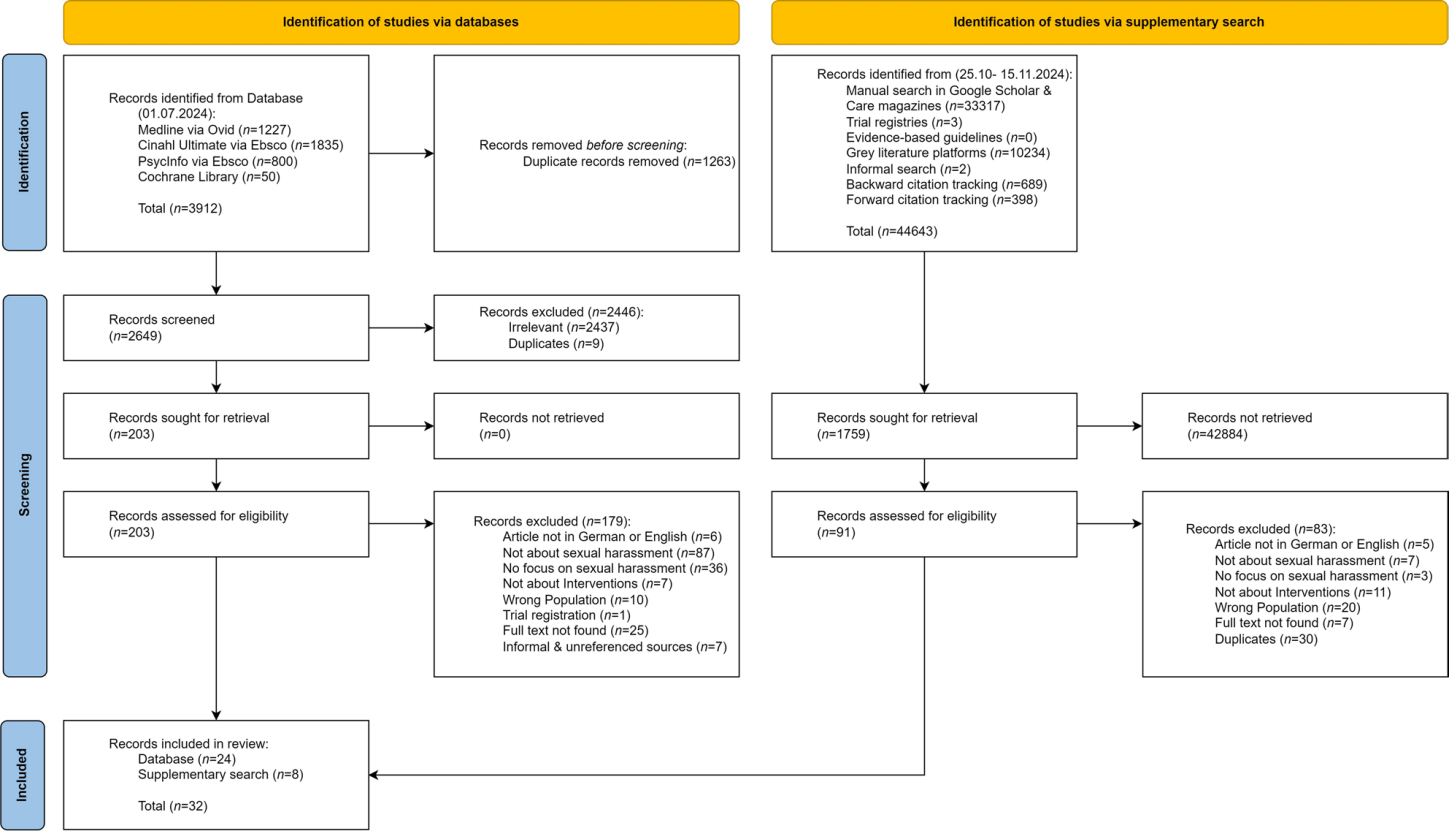
Flowchart of the searched, screened and included records. Note. During the supplementary search in Google Scholar and grey literature platforms, only the first 10 pages (approximately 100 records per search) were screened. ‘Not about sexual harassment’ = topic absent; ‘No focus on sexual harassment’ = mentioned but not analysed separately. n = Number of records

Through the electronic database searches, we identified a total of 3,912 records, while approximately 44,643 records were identified through the supplementary search. After deduplication, 2,649 records from the database search and 1,759 records from the supplementary search were screened for eligibility. Ultimately, 24 records from the database search and 8 records from the supplementary search were included, resulting in a total of 32 records (see Fig. 1). Inter-rater agreement for the screening process yielded a Cohen’s kappa of 0.741 for title and abstract screening and 0.675 for full-text screening.

### Record characteristics

Table 2 provides an overview of the included records. The 32 included records cover literature from 1984 to 2024. Approximately 40% of the included records (*n* = 12) followed the IMRAD structure. All IMRAD-structured records included empirical data and followed a scientific design. Among those not following the IMRAD structure (*n* = 20), only four records also included empirical data, while the rest were non-empirical works, including non-scientific methods, such as expert opinions, commentary papers, and guidelines. The records are diverse and encompass various study designs and record types, including expert opinions with a literature overview (*n* = 12), quantitative study designs (*n* = 10), review articles (*n* = 3), qualitative study designs (*n* = 2), dissertations (*n* = 2), a mixed study design (*n* = 1), a commentary (*n* = 1) and a guideline (*n* = 1).

**Table 2:** Overview of the included records and their characteristics

General information	Nursing Setting	Study design or record type	Capacity level	Approach domains 1–8	Approach testing status
Records following IMRAD (Introduction, Methods, Results, and Discussion) structure					
Kikuchi et al. – 2024 [54]; Japan	Home visit nursing	Quantitative (cross-sectional)	Organisational	1)|3)|4)|6)|7)	Applied in practice
Kim & Kim – 2023 [55]; USA	Acute care	Quantitative (cross-sectional)	Individual and organisational	5)|7)	Evaluated
Jenner et al. – 2022 [56]; Germany	Acute care	Qualitative (content analysis)	Individual and organisational	1)|2)|3)|4)|5)|6)|7)	Applied in practice
Zeighami et al. – 2022 [57]; Iran	Acute care	Qualitative (content analysis)	Individual and organisational	2)|5)|6)|7)|8)	Applied in practice
Chang et al. – 2021 [58]; Taiwan	University, department of nursing	Quantitative (RCT)	Individual	6)	Evaluated
Fallahi Khoshknab et al. – 2015 [59]; Iran	Acute care	Quantitative (cross-sectional)	Organisational	2)	Applied in practice
El-Ganzory et al. – 2014; [60]; Egypt	Acute care	Quantitative (quasi-experimental)	Individual and organisational	1)|3)|4)|5)|6)|7)	Evaluated
Arulogun et al. – 2013 [61]; Nigeria	Acute care	Quantitative (cross-sectional)	Individual and organisational	1)|2)|4)|5)|6)|7)	Recommended
Kamchuchat et al. – 2008 [62]; Thailand	Acute care	Quantitative and Qualitative (multi-method)	Individual	7)	Recommended
Cogin & Fish − 2007 [63]; Australia	Acute care	Quantitative (model testing)	Organisational	1)|3)|6)	Recommended
West et al. – 1995 [64]; USA	Not specified / nursing workplaces generally	Quantitative (cross-sectional)	Organisational	1)|2)|3)|4)|5)|6)	Recommended
Kettl et al. – 1993 [65]; USA	Psychiatric hospital	Quantitative (cross-sectional)	Individual and organisational	5)|6)|7)|8)	Evaluated
Records NOT following IMRAD (Introduction, Methods, Results, and Discussion) structure					
David – 2024 [66]; USA	Not specified / nursing workplaces generally	Expert opinions with literature overview	Individual and organisational	4)|5)|6)|7)	Recommended
Lee Valentine – 2023 [67]; USA	Not specified / nursing workplaces generally	Dissertation	Organisational	1)|3)|4)|5)|6)	Recommended
Kapoor & Grover − 2021 [68]; Canada	Not specified / nursing workplaces generally	Commentary	Organisational	3)|4)|6)	Recommended
Micale – 2021 [69]; USA	Acute care	Dissertation	Individual	6)	Evaluated
Uslu & Buldukoglu – 2021 [70]; Turkey	Not specified / nursing workplaces generally	Review article	Individual	5)|7)|8)	Recommended
Ross et al. – 2019 [71]; USA	Not specified / nursing workplaces generally	Expert opinions with literature overview	Individual and organisational	1)|2)|3)|4)|5)|6)|7)	Recommended
SBK – 2019 [72]; Switzerland	Not specified / nursing workplaces generally	Guideline	Individual and organisational	1)|2)|3)|4)|5)|6)|7)|8)	Recommended
Kane-Urrabazo – 2007 [73]; USA	Acute care	Expert opinions with literature overview	Organisational	2)|3)|4)|5)|6)	Recommended
Valente & Bullough – 2004 [74]; USA	Not specified / nursing workplaces generally	Expert opinions with literature overview	Individual and organisational	1)|2)|4)|5)|6)|7)	Applied in practice
Hamlin & Hoffman – 2002 [75]; Australia	Acute care	Review article	Individual and organisational	4)|5)|6)|7)	Recommended
Gardner & Jonson – 2001 [76]; USA	Not specified / nursing workplaces generally	Review article	Organisational	1)|2)|3)|4)|5)|6)	Recommended
Moore & Whitehead – 1999 [77]; USA	Acute care	Quantitative (cross-sectional)	Organisational	1)|3)|4)|5)|6)	Applied in practice
Davidhizar et al. – 1998 [78]; USA	Not specified / nursing workplaces generally	Expert opinions with literature overview	Individual and organisational	2)|3)|5)|6)|7)	Recommended
Kaye – 1996 [79]; USA	Acute care	Expert opinions with literature overview	Individual and organisational	1)|4)|5)|6)|7)	Recommended
Slagle King – 1995 [80]; USA	Acute care	Expert opinions with literature overview	Individual and organisational	1)|3)|4)|5)|6)|7)	Applied in practice
Neuhs – 1994 [81]; USA	Acute care	Expert opinions with literature overview	Organisational	1)|4)|5)|6)	Recommended
Childers-Hermann – 1993 [82]; USA	Not specified / nursing workplaces generally	Expert opinions with literature overview	Individual	4)|5)|7)	Recommended
Julius & DiGiovanni – 1990 [83]; USA	Acute care	Expert opinions with literature overview	Organisational	4)|5)|6)	Recommended
Heinrich – 1987 [84]; USA	Home visit nursing	Expert opinions with literature overview	Individual and organisational	3)|7)|8)	Recommended
Grieco – 1984 [85]; USA	Not specified / nursing workplaces generally	Expert opinions with literature overview	Individual and organisational	1)|2)|3)|5)|6)|7)	Recommended

Note. (1) ‘Approaches addressing organisational culture’, (2) ‘approaches adapting infrastructure and working environment’, (3) ‘approaches targeting leadership’, (4) ‘guidelines as approaches’, (5) ‘reporting of incidents as approaches, (6) ‘approaches in education’, (7) ‘nurses’ approaches to reacting or coping’, and (8) ‘patient-centred approaches’

Analysis of the ‘testing status’ revealed that most of the records (*n* = 20) reported approaches that were recommendations on the basis of input from participants, stakeholders, authors, or the previous literature, with no information on whether these approaches were applied in practice or evaluated in any way. Some records (*n* = 7) included approaches that were stated to be applied in practice, but without information on whether they had been evaluated. Only a few records (*n* = 5) reported on approaches that were evaluated for effectiveness or utility.

More than half of the records (*n* = 17) reported specifically on the acute care setting, followed by 34% of the records (*n* = 11) that did not specify a particular nursing setting but referred to nursing workplaces generally. Other settings included home visit nursing (*n* = 2), psychiatry (*n* = 1) and nursing university (*n* = 1). Although the latter is not a typical nursing workplace, it is a setting where nursing students in or following professional internships are prepared for workplace-related experiences of sexual harassment, thus aligning with the scope of this review. Most of the included records (*n* = 19) originated from the United States of America (USA).

### Description of the identified approaches

The analysis revealed a broad range of approaches to preventing sexual harassment of nurses or minimising its adverse consequences. Half of the records (*n* = 16) report approaches at both the individual and organisational levels, followed by those at the organisational level only (34.4%, *n* = 11) and, those at the individual level only (15.6%, *n* = 5). No approaches at the network level are reported.

**Fig. 2:**
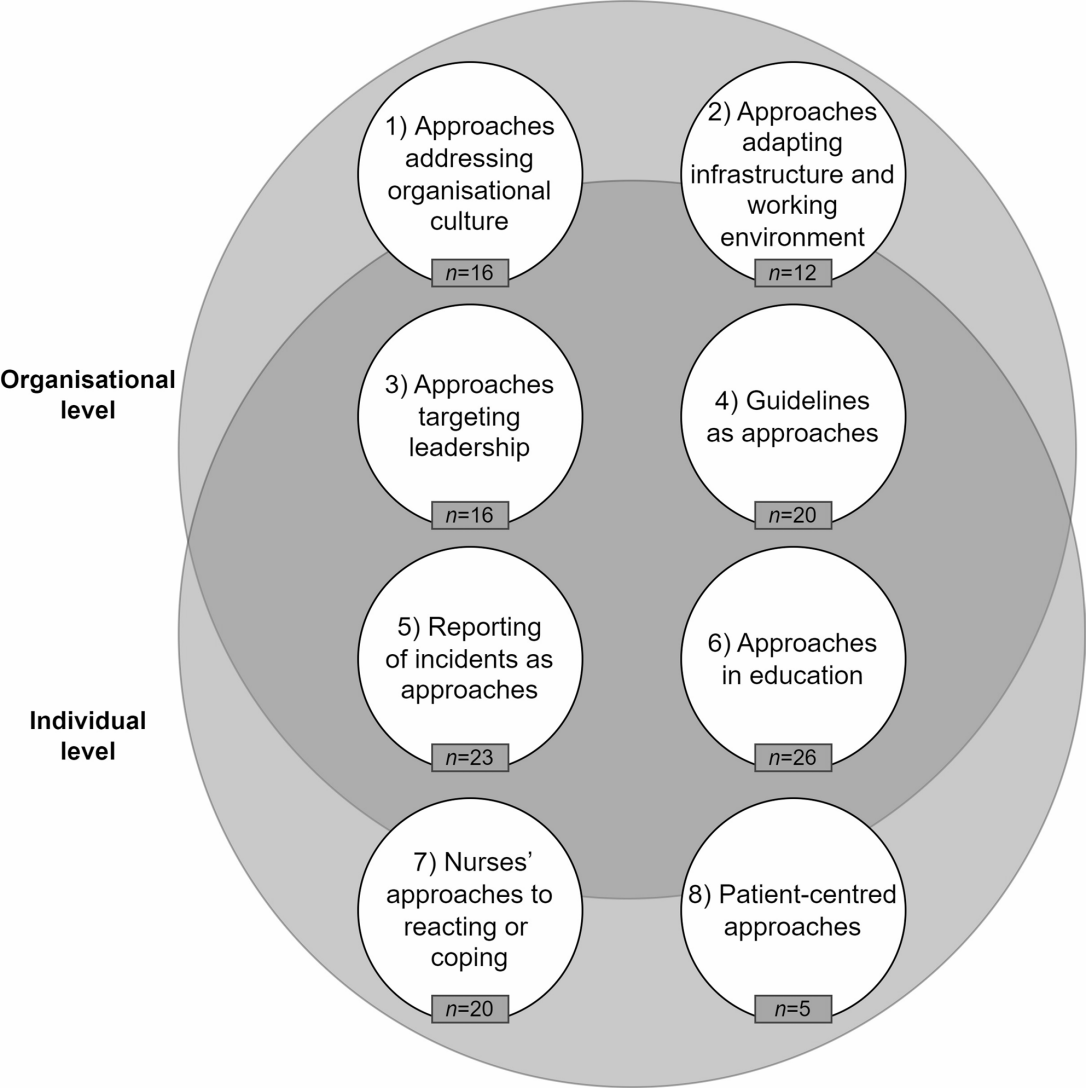
Overview 8 domains. Note. n = Number of records reporting approaches on the given domain

The approaches identified in the included records were categorised into eight domains and are described in the following subsections. Figure 2 provides a visual representation of the eight domains across the individual and organisational levels. Importantly, the domains do not belong exclusively to a single level, as they encompass approaches that may target both the individual and organisational levels. However, Fig. 2 illustrates which level is predominantly addressed within each domain.

***1) Approaches addressing organisational culture***

Half of the records (*n* = 16) describe approaches to create a culture that discourages sexual harassment of nurses [54, 56, 60, 61, 63, 64, 67, 71, 72, 74, 76, 77, 79–81, 85]. Neuhs (USA, 1994) [81] and Ross et al. (USA, 2019) [71] recommend enforcing an ethics-driven and respectful organisational culture that fosters values and standards to guide behaviour, decision-making, and relationships to counteract sexual harassment. Additionally, some records suggest addressing sexual harassment directly and transparently and eliminating the code of silence [71, 76, 80]. Doing so can be achieved through awareness raising and sensitisation [54, 79].

Seven of the included records mention zero tolerance policies as key to raising awareness and establishing healthy workplaces that are free of sexual harassment [56, 60, 67, 71, 72, 76, 77]. Moreover, strengthening cohesive work relationships may contribute to a work environment characterised by civility and kindness, where everyone is treated with dignity and respect and feels safe [63, 67, 71].

In particular, records propose creating opportunities to discuss workplace sexual harassment, such as during ward rounds or formal staff meetings [60, 72, 85]. Furthermore, the records indicate that organisational culture could be positively influenced by establishing rules to guide workplace behaviour [61, 74], with participants in the study by Arulogun et al. (Nigeria, 2013) [61] specifically recommending dress code regulations. Additionally, measures to strengthen gender equality, particularly in departments dominated by men, are suggested as a potential long-term strategy to protect against sexual harassment [56].

***2) Approaches adapting infrastructure and working environment***

One-third of the included records (*n* = 12) provide examples of how to improve work conditions and adapt the environment to counter sexual harassment [56, 57, 59, 61, 64, 71–74, 76, 78, 85].

Jenner et al. (Germany, 2022) [56] describe how positively influencing work conditions, such as addressing staff shortages, unclear schedules, and stress, can decrease the risk of inappropriate behaviour, including sexual harassment. Zeighami et al. (Iran, 2022) [57] suggest that changing nurses’ work schedules, patient assignments, or wards could also be helpful in cases of sexual harassment, for example, having female nurses care for female patients and male nurses care for male patients or combining young and experienced staff during each shift.

According to Fallai Khoshknab et al. (Iran, 2015) [59], Ross et al. (USA, 2019) [71] and Zeighami et al. (Iran 2022) [57], specific infrastructure adaptations, such as having security guards present and installing cameras, could influence the occurrence and handling of sexual harassment of nurses. Providing counselling services and hotlines for affected nurses or implementing buddy systems may help reduce the adverse consequences if sexual harassment occurs [56, 61, 73, 74, 78, 85]. In general, the appointment of a trusted person or a special investigation committee can help manage and respond effectively to sexual harassment [64, 72].

***3) Approaches targeting leadership***

According to 50% of the records (*n* = 16), management plays a crucial role in addressing sexual harassment of nurses [54, 56, 60, 63, 64, 67, 71–73, 76–78, 80, 84–86]. As highlighted by West et al. (USA, 1995) [64] and Kane-Urrbazo (USA, 2007) [73], tackling sexual harassment requires a proactive approach from leadership. Such an approach includes consistent and clear anti-harassment messages from those in authority [67].

Records indicate that (nurse) leaders can influence the occurrence of workplace sexual harassment of nurses as well as its adverse consequences by adapting their own behaviour. Managers, those in senior positions, and supervisors should act as role models by avoiding gender-related comments, demonstrating appropriate responses to toxic situations, and exhibiting protective and respectful leadership behaviour [56, 64, 71, 76, 77].

Those in authority should act as mediators on behalf of those affected and must make it clear that incidents of harassment will not be tolerated [56, 78]. Prompt and remedial actions by leaders are needed to protect nurses [73, 77]. Victims should never be blamed for being sexually harassed, and leaders should be especially cautious if a nurse has been a victim multiple times [54, 72, 84]. Records agree on leadership’s duty to recognise sexual harassment, support nurses during incidents, document incidents, and conduct follow-up and monitoring of the procedures taken [56, 67, 72, 73, 77, 78, 80, 84, 85]. Furthermore, leadership is responsible for conducting regular evaluations of the work environment, identifying possible dangers to nurses or high-risk areas, and charting the frequency of sexual harassment incidents [57, 67, 84–86].

El-Ganzory et al. (Egypt, 2014) [60] emphasise the need to reflect on and reconsider nursing theory and practice. Nursing care often focuses on the nurse‒patient relationship, which can obscure broader social issues such as gender discrimination and sexual harassment [60]. Addressing gender differences and promoting women to leadership positions are additional strategies mentioned at the leadership level to combat workplace sexual harassment in nursing [63, 67].

***4) Guidelines as approaches***

Effective, clear, and binding guidelines or policies against sexual harassment are highlighted as crucial in more than 60% (*n* = 20) of the included records [54, 56, 60, 61, 64, 66, 67, 71–77, 79–83, 86]. In addition, Jenner et al. (Germany, 2022) [56] mentioned the need for specific guidelines for patients, including a code of conduct.

Not only is having health care guidelines considered important, but nurses must also be educated in them [56, 60, 66, 71, 73, 76, 79, 80, 82]. Having guidelines in a tangible form, for example, as pocket cards, can help raise awareness and increase accessibility [66, 71, 82].

Several of the included records elaborate on the components of effective guidelines, suggesting that guidelines should define sexual harassment [74, 83], highlight rights and responsibilities [64, 71, 74], and clarify disciplinary actions [56, 61, 67, 71–74, 80]. Examples of disciplinary actions include bans from the clinic, jail terms, and verbal or written reprimands [56, 61, 67].

***5) Reporting of incidents as approaches***

Reporting of incidents is deemed important for combating sexual harassment of nurses in almost three-quarters (*n* = 23) of the included records [55–57, 60, 61, 64–67, 70–82, 85]. Whether through a formal complaint [75, 79, 82], through a standardised process [55, 75, 76, 80], or by verbally informing a coworker or supervisor [74, 78], reporting incidents is crucial for preventing new incidents. Incident documentation should include details of what occurred, when, where, who was involved, and any possible measures taken in the situation [64, 66, 70, 73, 75, 77–82]. Following a report, appropriate persons should conduct a timely, confidential inquiry and gather information from all involved parties [77, 78].

Six records emphasise the importance of informing nurses about reporting processes [74, 78, 80, 81] and providing easy procedures to encourage reporting [75, 76]. Furthermore, records stress that all complaints must be taken seriously and treated as a priority to minimise adverse consequences [57, 64, 72, 78, 85]. Gardner & Johnson (USA, 2001) [76] highlight the importance of maintaining confidentiality throughout the reporting process. They conclude that the greater the degree of confidentiality is, the more comfortable nurses are in reporting incidents of sexual harassment.

***6) Approaches in education***

Education on sexual harassment is the most frequently mentioned approach to combating sexual harassment of nurses, reported by 81.3% (*n* = 26) of the included records [54, 56–58, 60, 61, 63–67, 69, 71–81, 83, 85, 86]. Education and training for nurses help to raise awareness [54, 66], and therefore, to recognise, predict, prevent, intervene in and cope with sexual harassment [58, 65, 69, 81]. The effectiveness of educational measures in terms of enhancing knowledge about sexual harassment, coping behaviours, empowerment, self-efficacy, and emotional responses was examined and confirmed in four empirical records [58, 60, 65, 69].

Records recommend educating nurses not only once, such as during nursing school, but also repeatedly in training sessions annually or with each new employment [57, 58, 71, 76, 78, 85]. Some records suggest training specifically for nurses [60, 65, 66, 75, 78, 81, 85] and supervisors [56, 64, 79–81], whereas others advocate for training all staff [64, 67, 73–76, 83]. In addition to educating staff, three records emphasise the importance of educating patients on what constitutes sexual harassment, as well as their rights, expectations, and obligations [63, 71, 72].

Specific examples of methods for implementing education programmes can be found throughout the records. These include webinars, multimedia eBooks, guideline programmes, simulation-based training, role-playing, self-protection training, de-escalation training, and conflict management [56, 58, 60, 69, 75, 81, 85, 86].

***7) Nurses’ approaches to reacting or coping***

Approaches for nurses to react to or cope with sexual harassment were reported in 62.5% (*n* = 20) of the records [54–57, 60–62, 65, 66, 70–72, 74, 75, 78–80, 82, 84, 85]. These approaches were diverse and varied by context and perpetrator. The approaches mentioned within the single records were categorised into six sub-domains: (a) risk assessment, (b) direct confrontation, (c) protective behaviour, (d) seeking help, (e) self-reflection and (f) coping.

**a) Risk assessment**

Risk assessment tools can be used by nurses to evaluate the risk of violence or assess their work environment. The use of such tools helps nurses choose the appropriate strategy according to the context [54, 55, 84]. Kim & Kim (USA, 2023) [55] tested the utility of a risk assessment tool to recognise, grade and report various types of workplace violence, including sexual harassment. They concluded that the tool is relevant and useful for nurses to recognise and report various types of workplace violence.

**b) Direct confrontation**

If nurses adopt a prompt and direct approach to incidents of sexual harassment, they clearly communicate that such behaviour is unacceptable. This approach was documented in nearly half of the records (*n* = 15) and encompasses verbal, nonverbal, or physical methods to halt the harassment [56, 60, 61, 65, 66, 70–72, 74, 75, 78–80, 82, 84]. Examples of direct confrontation reactions include verbally or nonverbally telling the perpetrator to stop [56, 60, 65, 66, 70, 75, 79, 80], confronting the unacceptable behaviour [56, 60, 61, 72, 75, 78–80, 82, 84], writing a letter to the perpetrator [79, 82], verbally redirecting the conversation [65], requesting a professional relationship [79], defining roles and setting limits [56, 65, 72, 74], speaking out using proven phrases [66, 72, 75, 80, 82], scolding the perpetrator [60], and physically stopping or fighting the perpetrator [60, 61, 65].

**c) Protective behaviour**

Eight records describe approaches used by nurses that include avoidance and distancing to protect themselves from situations or people who may cause sexual harassment [56, 57, 60, 61, 65, 66, 71, 84]. If nurses use avoidance as a strategy, they remain alert, are careful and actively avoid specific situations [57, 60, 65]. Furthermore, nurses avoid being too friendly to prevent their behaviour from being misinterpreted [57] or avoid physical contact [65], and they do not accept gifts [65], ignore sexual harassment [56, 84] or change topics [60] and remain silent [60].

Distancing goes one step further than avoidance. Here, nurses actively distance themselves physically from the perpetrator or the location [56, 60, 61]. Examples described in the records for distancing behaviour include changing patient assignments, shift work, or wards to avoid sexual harassment [57, 71], stepping away [71], keeping a safe distance [57], leaving the situation [66], breaking off the relationship with the perpetrator [61], or withdrawing from the place, perpetrator, or service [56, 60, 61, 84].

**d) Seeking help**

Seeking help is an approach reported in eight records and is used by nurses when they decide not to handle a situation alone [56, 62, 71, 72, 75, 79, 82, 85]. In this approach, nurses call for help from peers [56, 71, 85] and ask to be accompanied by a second person during close contact [62]. Additionally, nurses may form alliances [56] and seek other victims or witnesses [75, 82]. Contacting higher authorities and seeking legal advice are further examples of obtaining help [72, 79, 82].

**e) Self-reflection**

Six records described approaches in which nurses engage in active self-reflection [57, 65, 72, 75, 80, 84]. Nurses evaluate their own behaviour [84], step away from the victim role [75], review their actions [84], consider the appropriateness of work clothing [57, 72], their choice of words [65, 72], or if they overstepped a boundary [65]. In addition, nurses take care to communicate in a clear and explicit manner, avoiding ambiguity, misunderstandings and discussions about personal matters [65, 72, 80].

**f) Coping**

Approaches that nurses use to cope with experiences of sexual harassment and to minimise its adverse consequences were referred to in four records [60, 61, 66, 70]. Possible coping measures included avoiding thinking or talking about sexual harassment [60], using distractions to deal with remorse [61], practising self-care [66], being aware of one’s own emotions [70], and taking sick leave [60].

***8) Patient-centred approaches***

Patient-centred approaches are a special set of strategies for nurses dealing with sexually harassing patients. These approaches consider that, behind harassing behaviour, there might be an unfilled patient need. If that need is considered, it can prevent patients’ sexual harassment of nurses. Only 15.6% of the included records (*n* = 5) address this perspective [57, 65, 70, 72, 84], without directly drawing on patient data. Patient-centred approaches include actively involving patients in care planning and informing them about their rights and responsibilities [70, 72]. According to Uslu & Buldukoglu (Turkey, 2021) [70] such actions help reduce fear and loss of power.

Heinrich (USA, 1987) [84] and Uslu & Buldukoglu (Turkey, 2021) [70] suggest that, before judging a patient, it is beneficial to explore possible motives and to assess the underlying context or illness situation. Identifying patients’ needs, allowing personal items, and giving them privacy can improve their comfort and well-being [57, 65, 70, 72]. For some patients, it may be necessary to place them in a single-occupancy room or to use signs that remind others of private environments [70]. Kettl et al. (USA, 1993) [65] emphasise the importance of discussing sexuality with patients and educating them. In addition, providing positive feedback can help strengthen appropriate behaviour [70].

## Discussion

### Summary of evidence

The aim of this scoping review was to identify approaches to combating sexual harassment of nurses or its adverse consequences at different levels. The 32 included records provide a wide range of different approaches used to prevent sexual harassment and minimise its consequences. The approaches were categorised into 8 domains, with ‘approaches in education’ being the most frequent, followed by ‘reporting of incidents as approaches’. The third most frequently mentioned were the ‘guidelines as approaches’ and ‘nurses’ approaches to reacting or coping’ domains. ‘Approaches addressing organisational culture’ and ‘approaches targeting leadership’ were in fourth place. ‘Approaches adapting infrastructure and work environment’ and ‘patient-centred approaches’ were the least frequently mentioned. All the approaches described in the records are at the individual and organisational levels. The broad inclusion criteria of this scoping review, encompassing both scientific and non-scientific sources, were intentionally applied to comprehensively map the current state of the literature and the types of evidence available. Ultimately, many of the approaches identified are recommended or applied in practice rather than empirically evaluated. Thus, some approached may not be effective, and others may even be harmful, highlighting the need for future research to test and validate more promising approaches.

The included records comprise 40 years of literature. Approximately 60% of the records are 10 years old or older, do not follow the typical IMRAD structure and are mainly of non-scientific methods. The reason may be that sexual harassment has been considered a form of gender discrimination and legally actionable in the USA only since the late 1970s and early 1980s [87, 88]. The USA was among the first countries to legally define sexual harassment as a form of workplace discrimination, with other nations subsequently following this example [89, 90]. Although the number of countries that have enacted legislation prohibiting sexual harassment increase each year, legal frameworks still vary considerably across nations [90]. The relatively recent and evolving recognition of sexual harassment of nurses may explain why much of the evidence comes from less rigorous scientific designs or non-scientific methods, as the issue needed initial exploration and definition. An additional explanation may be that the topic has not yet been prioritised by policy-makers or the research community and has received less attention than other types of violence [2, 91]. Despite the attention drawn by the #MeToo and #EndNurseAbuse movements, sexual harassment is often not taken seriously [91–93]. Nursing research funding is mostly directed towards well-established areas with high visibility and perceived impact, such as oncology, geriatrics, and paediatrics [94, 95]. These are often seen as safer investments, whereas sensitive and politically charged topics such as sexual harassment receive comparatively little support [95]. Furthermore, the focus mostly lies on patients and their disease rather than on nurses’ working conditions, even though safe and supportive work environments are essential for high-quality patient care [94]. Difficulties in securing funding for such topics may partly explain the limited empirical research and the resulting scarcity of systemically developed and rigorously evaluated interventions.

The majority of the included records originate from acute care settings. This predominance should be considered when interpreting the findings, as approaches may not fully reflect the needs of other care contexts, such as long-term care or home visiting nursing. This underscores the need for further research in other care settings. One possible explanation for this predominance is that acute care settings, particularly emergency and psychiatric wards, have been more frequently examined in workplace violence research [96, 97], and some evidence suggests higher rates of sexual harassment in these contexts [2, 4, 98].

Despite the aim of identifying approaches at all three levels (individual, organisational, and network), no strategies at the network level were found. This absence is noteworthy, as Mirzoev et al. [33] emphasise that addressing all three levels is essential for achieving long-term, sustainable change in health systems. While general governmental policies and guidance on workplace sexual harassment exist in many countries (e.g., Australia, the USA, and Switzerland [99–101]), these documents are typically not specific to the nursing profession and therefore did not meet the inclusion criteria of this review. As previously noted, the relatively recent recognition of sexual harassment as a significant issue in nursing may again be a contributing factor to the absence of network-level approaches. Moreover, network-level strategies could also have been neglected owing to their complexity and need for broader collaboration. As Mirzoev et al. [33] highlight, developing network-level interventions requires a combination of individual and organisational expertise, along with network-specific attributes such as shared goals, decision-making structures, resource access, and effective communication. These factors make the development and implementation of network level interventions particularly challenging. Nevertheless, given their importance, potential approaches for nurses at the network level are needed. Such approaches could include social media initiatives such as #EndNurseAbuse, which was launched by the American Nurses Association (ANA). According to the ANA, supporting these initiatives promotes accountability for workplace abuse, harassment, and inequality [102].

The analysis reveals that nearly 60% of the included records originate from the USA. One reason for the predominance of the USA might be its proactive stance in addressing workplace sexual harassment through laws [87, 88]. Additionally, the USA has played a leading role in the feminist movement and the initiation of the #MeToo movement, which brought attention to workplace sexual harassment [103, 104]. According to the review by Spector et al. [2], the rates of sexual harassment were the highest in the Anglo region. This finding is attributed to cultural differences in sensitivity to sexual harassment, with presumably more advanced awareness and reporting in the Anglo region [2]. Even though records from various other countries are also represented in the review, the predominance of records from the USA could limit the transferability of the approaches mentioned to other geographical regions.

This review did not aim to analyse or compare cultural or regional differences across the included records. However, cultural context plays an important role in both the interpretation and applicability of the identified approaches. Research has shown that the prevalence, perception, and consequences of sexual harassment can vary significantly across different countries and settings [1, 2, 25, 98, 105]. For instance, recommendations on professional conduct, professional appearance, interpersonal boundaries, and individual behaviour may be shaped by local norms and expectations, leading to different interpretations across cultural contexts [59, 62, 63, 78, 86, 98]. Consequently, not all approaches may be equally appropriate or effective in every context. When applying these findings, policymakers, health care organisations and nurses should therefore consider cultural sensitivity and contextual adaptation to ensure that interventions are both applicable and acceptable. Further research is needed to explore how cultural and legal frameworks influence the implementation and success of approaches.

All the records included agree that sexual harassment of nurses is a problem that needs to be addressed. This finding is consistent with existing systematic reviews on this topic [1, 25, 98, 105]. The records propose various approaches to addressing this problem. Some of these approaches have attracted attention because they are innovative, ethically critical, mentioned frequently, or conflicting.

One approach that can be seen as both innovative and ethically critical is mentioned in the category ‘approaches adapting infrastructure and working environment’. Zeighami et al. (Iran, 2022) [57] reported that installing cameras in various locations prevented sexual harassment. When nurses pointed out the cameras to the perpetrator, the behaviour stopped immediately [57]. A 2023 newspaper article from England by Dean [106] reported on a hospital testing body cameras in its emergency department, similar to those widely used by paramedics and police. The cameras did not record but displayed real-time footage on a screen, prompting self-reflection and stopping harassment. Nurses reported feeling safe and supported wearing those cameras. While most nurses support the long-term use of cameras, ethical concerns emerge. Body cameras can invade privacy during intimate procedures and reinforce power imbalances [106], which in turn can increase the risk of sexual harassment [15, 70, 72].

The category most frequently mentioned in this scoping review was ‘approaches in education’, which aligns with the findings of Kumari et al. [34]. Education was also the only approach where effectiveness was examined. Chang et al. (Taiwan, 2021) [58], Kettl et al. (USA, 1993) [65], El-Ganzory et al. (Egypt, 2014) [60], and Micale (USA, 2021) [69] reported improved knowledge, coping, empowerment, self-efficacy, and emotional responses in regard to sexual harassment in pre-post comparisons following an educational intervention.

Additionally, ‘reporting of incidents as approaches’ was stated as important in most records. The reporting of incidents is very important because employers can fulfil their duty to protect employees from sexual harassment only if they are aware of such incidents [88]. However, reporting incidents itself proves to be challenging, as underreporting is a major issue [2, 16, 29, 31, 38, 66]. The records highlight the need for a simple, confidential tool with training and guidance on what to document [55–57, 60, 61, 64–67, 70–82, 85]. The importance of well-designed and standardised reporting systems is also stressed by Fenwick et al. [107] and Odes et al. [108].

Approaches that can be seen as conflicting are mentioned between the ‘approaches addressing the organisational culture’, ‘approaches targeting leadership’ and ‘nurses’ approaches to reacting or coping’ domains. Although records agree on zero tolerance against sexual harassment and that nurses should not be blamed for being sexually harassed [56, 66, 67, 71, 72, 76–78, 84], some approaches suggested that nurses’ own behaviour or clothing can lead to sexual harassment [57, 61, 65, 72, 74, 75, 80, 84]. Records propose using self-reflective behaviour [57, 65, 72, 75, 80, 84] or regulations on dress codes, to counteract [61, 74]. For example, some approaches include being mindful of word choice, avoiding discussions of personal sexuality, and refraining from wearing tight clothing or using cosmetics [57, 65, 72, 84]. In line with these approaches, Watts [109] argues in a reader’s letter published in England in 1993 that nurses must also take responsibility when being sexually harassed. Nurses should ensure that their actions do not send signals that encourage sexual harassment [109]. Although Watts’ [109] view may include some valid points, it reflects the discourse of its time, written more than 30 years ago. While nurses too often blame themselves for incidents of sexual harassment [15, 16], current understandings emphasise the importance of avoiding victim blaming [72, 110]. Victim blaming can exacerbate psychological harm, discourage reporting, and hinder access to support services, and should therefore be avoided [110].

Most records take the perspective of nurses and their need for protection against sexual harassment. Very few records reflect on patients’ needs for privacy, sexuality, and control from a theoretical or conceptual perspective. Although the review did not aim to analyse approaches by perpetrator group, patient-centred views stood out due to their distinct framing and differing perspective. These ‘patient-centred approaches’ are worth considering when nurses are faced with patients’ sexual harassment. Nevertheless, ethical reflection is needed, considering the boundaries of a nurse’s duties and comfort. For example, Kettl et al. (USA, 1993) [65] state that nurses should listen to patients’ sexual concerns and provide education about sexuality. However, the line between expressing sexual desires and attempting to seduce can be very fine [84]. Furthermore, discussing sexuality can cause patients to feel shame and a loss of control, potentially leading to harassing behaviours as a way to rebalance power [70, 72]. Zeighami et al. (Iran, 2022) [57] advise respecting patients’ privacy and avoiding situations that could lead to sexual harassment. While nurses should create opportunities for patients to discuss their sexuality as a care intervention [111], they must remain aware of their own emotions and boundaries, recognising their right to distance themselves if a patient’s sexual needs or behaviours are inappropriate [56, 60, 61, 70]. In addition, in their systematic review, Fennell and Grant [111] recommend specifically training nurses on how to provide sexual health care education for patients, so that they feel confident and comfortable providing such care interventions. Whether it is appropriate to discuss sexuality with a patient depends very much on the situation and should be left to the nurse to decide whether it is permissible in the situation.

The persistently high rate of sexual harassment of nurses [3, 4, 7, 25, 32, 98] suggests that single approaches are insufficient [63]. Multiple proactive and reactive measures at different levels need to be implemented and evaluated [30, 33, 38, 39]. There are many approaches available in the literature to counteract sexual harassment of nurses or to minimise its adverse consequences. Which approach is best depends on the situation and context [56, 84]. Although their effectiveness has not yet been fully examined or confirmed, this fact should not deter nurses or health care organisations from acting. In general, sexual harassment is not to be accepted as part of the job, and nurses are not to blame for it [66].

### Limitations

This scoping review had several limitations. For example, the full texts of 32 records were not found despite extensive searches, or they were not accessible with reasonable effort; therefore, they were excluded. For pragmatic reasons, only records in English and German were included. Reporting bias may therefore be present. This language restriction may also have limited the inclusion of studies from certain cultural contexts, thereby affecting the representativeness of the findings across different cultures. A further methodological limitation may be that the supplementary search was conducted by only one researcher, with full-text verification by a second reviewer. Additionally, the data extraction procedure was performed by a single researcher, which may introduce researcher bias. To address these limitations, regular meetings and discussions within the project team were held to review the methodology and to critically discuss key decisions during data extraction.

Another limitation is the deviation from the protocol due to the abundance of literature based on non-scientific methods. As a result, the research team excluded sources such as newspaper articles and expert opinions that lacked reference to any empirical or non-empirical research. Nonetheless, the development of a protocol and the implementation of systematic processes for identifying, extracting, and analysing relevant records can be considered a methodological strength of this study. Despite these limitations, this scoping review is the first to map possible approaches to combating sexual harassment of nurses, and it has generated valuable results.

## Conclusions

This scoping review identified a range of approaches to combating sexual harassment of nurses or its adverse consequences at the individual and organisational levels. However, no approaches were identified at the network level. Moreover, research on the effectiveness and feasibility of these approaches in practice is still lacking. Addressing workplace sexual harassment of nurses requires approaches on multiple levels. Therefore, empirical research is needed to address the lack of systematically developed and rigorously evaluated approaches to counter sexual harassment of nurses across all levels. Future studies should evaluate the effectiveness and feasibility of existing individual and organisational approaches, as well as develop and test network level approaches that promote interorganisational collaboration for sustainable change.

The results of this scoping review may guide the development, implementation, and evaluation of comprehensive strategies to prevent sexual harassment of nurses or to minimise its adverse consequences on multiple levels. Additionally, overarching recommendations and guidelines could be developed to assist organisations in creating such concepts. To systematically evaluate the approaches identified in this scoping review within different contexts, further research is needed.

## Supplementary Information

Below is the link to the electronic supplementary material.

Supplementary Material 1

## Data Availability

No datasets were generated or analysed during the current study.

## Abbreviations

IMRAD: Introduction, Methods, Results, and Discussion
JBI: Joanna Briggs Institute
LLMs: Large Language Models
PCC: Population, Concept, Context
RCT: Randomised controlled trial
SBK: Swiss Association of Nurses (Schweizer Berufsverband der Pflegefachfrauen und Pflegefachmänner)
USA: United States of America
